# Metabolic Landscape of Bronchoalveolar Lavage Fluid in Coronavirus Disease 2019 at Single Cell Resolution

**DOI:** 10.3389/fimmu.2022.829760

**Published:** 2022-03-08

**Authors:** Ming-Ming Shao, Meier Shi, Juan Du, Xue-Bin Pei, Bei-Bei Gu, Feng-Shuang Yi

**Affiliations:** ^1^ Department of Respiratory and Critical Care Medicine, Beijing Institute of Respiratory Medicine and Beijing Chao-Yang Hospital, Capital Medical University, Beijing, China; ^2^ Eberly College of Science, The Pennsylvania State University, University Park, PA, United States; ^3^ Department of Emergency Medicine, Beijing Chao-Yang Hospital, Capital Medical University, Beijing, China; ^4^ Department of Anesthesia, Sichuan Academy of Medical Sciences & Sichuan Provincial People’s Hospital, Academy of Sciences Sichuan Translational Medicine Research Hospital, Chengdu, China

**Keywords:** Metabolic reprogramming, cytokine storm, COVID-19, single cell RNA sequencing, bronchoalveolar lavage fluid

## Abstract

Abnormal function of immune cells is one of the key mechanisms leading to severe clinical symptoms in coronavirus disease 2019 patients, and metabolic pathways can destroy the function of the immune system by affecting innate and adaptive immune responses. However, the metabolic characteristics of the immune cells of the SARS-CoV-2 infected organs *in situ* remaining elusive. We reanalyzed the metabolic-related gene profiles in single-cell RNA sequencing data, drew the metabolic landscape in bronchoalveolar lavage fluid immune cells, and elucidated the metabolic remodeling mechanism that might lead to the progression of COVID-19 and the cytokine storm. Enhanced glycolysis is the most important common metabolic feature of all immune cells in COVID-19 patients. CCL2^+^ T cells, Group 2 macrophages with high SPP1 expression and myeloid dendritic cells are among the main contributors to the cytokine storm produced by infected lung tissue. Two metabolic analysis methods, including Compass, showed that glycolysis, fatty acid metabolism, bile acid synthesis and purine and pyrimidine metabolism levels of CCL2^+^ T cells, Group 2 macrophages and myeloid dendritic cells were upregulated and correlated with cytokine storms of COVID-19 patients. This might be the key metabolic regulatory factor for immune cells to produce large quantities of cytokines.

## Introduction

According to statistics from the World Health Organization (WHO), as of June 18, 2021, coronavirus disease 2019 (COVID-19), caused by severe acute respiratory syndrome coronavirus 2 (SARS-CoV-2), has caused more than 170 million infections and 3.8 million deaths. SARS-CoV-2 infection is characterized by a broad spectrum of clinical symptoms, namely, asymptomatic disease, mild to moderate flu-like symptoms, severe pneumonia, acute respiratory distress syndrome, multiple organ dysfunction and even death (1, 2).

Studies have shown that aberrant immune cell function is one of the key mechanisms that cause severe clinical symptoms in COVID-19 patients (3–5). Single-cell RNA sequencing (scRNA-seq) technology can help researchers fully understand the changes in the immune response during the development of the disease. There are currently a number of scRNA-seq studies on COVID-19 (6–10) that provide us with important cellular and molecular insights, such as the downregulation of HLA class II expression of monocytes in COVID-19 patients (11), lymphopenia (12), immunity cell exhaustion (13), and increased levels of inflammatory cytokines (14). These studies often focus on changes in the dynamic ratio of immune cells in peripheral blood mononuclear cells (PBMCs) or bronchoalveolar lavage fluid (BALF) of COVID-19 patients and changes in inflammatory characteristics.

In recent years, many studies on immune metabolism have focused on the interaction between the immune response and cellular metabolism. For example, the pro-inflammatory cytokine IL-6 can regulate glucose and lipid metabolism, and the metabolite itaconate has been identified as a regulator of the cytokines IL-6 and IL-12, indicating that inflammatory factors play an important role in metabolic reprogramming (15, 16). In viral infectious diseases, metabolic pathways can regulate the innate and adaptive immune response of the host, and the metabolic demands caused by infection may exacerbate host cell stress, thereby disrupting homeostasis and triggering cell death and inflammation (17). For example, glucose metabolism plays an important role in regulating the cytokine storm induced by influenza A virus (18). The decrease of TCA activity can be observed in patients infected with yellow fever virus (19). Although there have been some studies on the characteristics of PBMCs and serum metabolism in COVID-19 patients (19–21), a more in-depth analysis of the metabolic characteristics of bronchoalveolar immune cells in COVID-19 patients and its association with disease progression are still unclear. As the characteristics of systemic immunity are different from those of the bronchoalveolar compartment, our understanding of cell-specific immune cell metabolic programming in COVID-19 patients is limited by the fact that PBMCs were used in most studies.

In this study, we reanalyzed scRNA-seq data of BALF samples from 9 COVID-19 patients (3 moderate cases and 6 severe cases) and 3 healthy controls (HCs) to identify the metabolic reprogramming of immune cells in the bronchoalveolar compartment and the correlation with disease progression. We drew the metabolic landscape of BALF immune cells from COVID-19 patients and identified several metabolic pathways and genes related to disease and inflammation, thereby gaining insight into metabolic mechanism of bronchoalveolar immune cells during SARS-CoV-2 infection.

## Materials and Methods

### Data Collection

BALF scRNA-seq data of 3 HCs (P51, P52 and P100) and 9 COVID-19 patients, who were enrolled from the Shenzhen Third People’s Hospital, were downloaded from the GEO database (https://www.ncbi.nlm.nih.gov/gds/, GSE145926) (22). Disease severity was defined as moderate (P141, P142, and P144) or severe (namely, severe [P143] and critical [P145, P146, P148, P149, and P152]). scRNA-seq data of nasopharyngeal swabs from 19 COVID-19 patients (8 moderate cases and 11 severe cases) and 5 HCs enrolled from a dual-center cohort from the Charité-Universitätsmedizin Berlin and the University Hospital Leipzig were downloaded from the Magellan COVID-19 data explorer at https://digital.bihealth.org (8).

### Single Cell Filtering, Clustering, Dimensionality Reduction and Visualization

Seurat (4.0.1) was used for subsequent calculations of the downloaded count matrix in R (4.0.2). If the number of expressed genes was <200 or >6,000, the UMI count was <1,000, and/or the mitochondrial gene percentage was >0.1, cells were removed. The “IntegrateData” function was used to remove the batch effect across different samples. The “NormalizeData” and “ScaleData” functions were used to standardize and normalize the matrix for subsequent cell clustering and dimensionality reduction. The top 2,000 highly variable genes identified by the “FindVariableFeatures” function were used in the “RunPCA” function to perform principal component analysis. The “FindClusters” function was used to cluster the cells with a resolution of 1.5. The “RunUMAP” and “TsnePlot” functions were used to project the cells into a two-dimensional space and visualize them. The “FindAllMarkers” function was used to identify differential expression genes (DEGs) compared with all other clusters. DEGs were defined as follows: detected in at least 25% of the cells, *P <*0.05 was based on the Wilcoxon rank sum test and Bonferroni correction, and |log_10_(fold change)| >0.3. Cell clusters were labeled through known classic markers.

### Reclustering of T Cells and NK Cells

T cells and NK cells were reclustered together. The “SubsetData” function was used to identify the T cells and NK cells for secondary cluster analysis with parameter “do.clean” setting to true. The calculation of matrices was repeated as described above, and data standardization, normalization, scaling, dimensionality reduction, and clustering were performed.

### Signaling Pathway Score

Subsequent pathway analysis was only performed on cell clusters that existed in both HCs and COVID-19 patients. The cytokine and inflammatory signaling pathways related to COVID-19 infection were collected based on the past literature (10), and the metabolism-related signaling pathways (nucleic acid metabolism, 4 pathways; amino acid metabolism, 14 pathways; lipid metabolism, 19 pathways; energy metabolism, 4 pathways; vitamin metabolism, 7 pathways; carbohydrate metabolism, 15 pathways) were collected from the Gene Ontology (GO) and Kyoto Encyclopedia of Genes and Genomes (KEGG) databases (**Supplementary Figure 1**). The “AddModuleScore” function in Seurat was used to evaluate signaling pathway scores and the Mann–Whitney rank test was used for statistical analysis.

### Correlation Analysis Between Metabolic Pathways and Cytokine or Inflammatory Signaling

The correlation between the metabolic pathway and the cytokine or inflammatory signaling of each cell cluster was evaluated by the score calculated above and the “cor” and “cor.test” functions in R.

### Compass Analysis

We followed Wagner et al. article for Compass analysis and defined core metabolic reactions based on the reaction metadata contained in the Recon2 database (23). Compass is an algorithm that uses single-cell transcriptomics data to characterize the metabolic state of cells with single-cell resolution and comprehensive network coverage. We performed the Wilcoxon rank sum test on the Compass score of each meta-response to calculate the statistical significance, and used Cohen’s d statistic to further evaluate the effect.

## Results

### Metabolic Reprogramming of BALF Immune Cells in COVID-19 Patients

**Figure 1:**
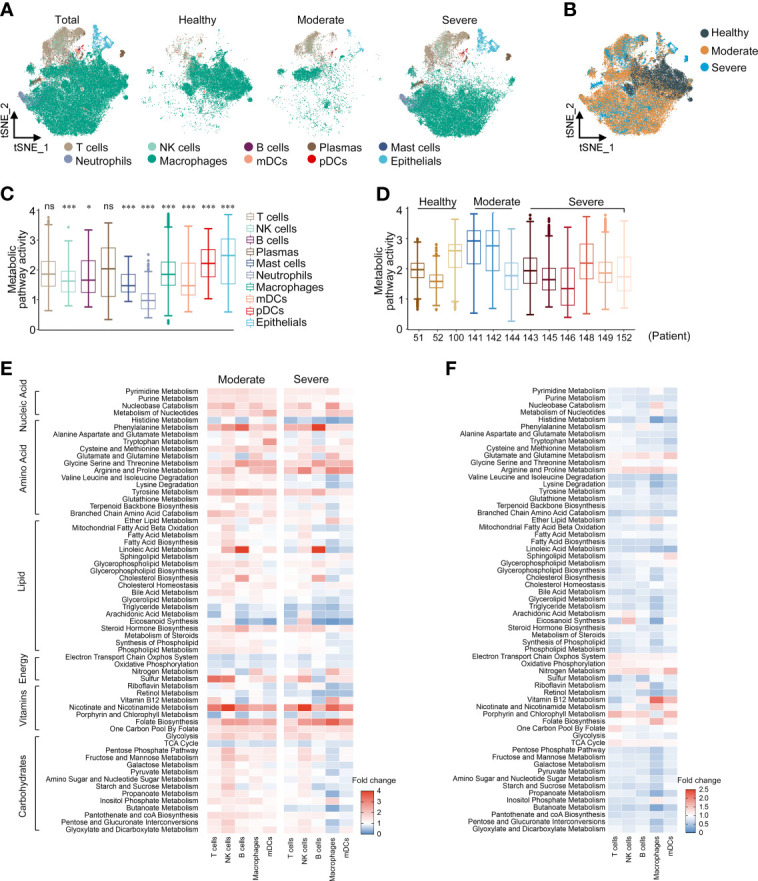
Metabolic reprogramming of immune cells in COVID19 patients. **(A)** tSNE plots of metabolic gene expression profiles within each sample type, color-coded by cell types. **(B)** tSNE plots of metabolic gene expression profiles, color-coded by sample type. **(C)** Distributions of metabolic pathway activities in different cell types. The box plots were defined by the interquartile range (IQR, the range between the 25 and 75%) and the median, whiskers represent the upper and lower value within 1.5 times the IQR. The significance was performed by Wilcoxon rank sum test. *P < 0.05, ***P < 0.001, ns, not significant. **(D)** Distributions of metabolic pathway activities in different patients. The box plots were defined by the interquartile range (IQR, the range between the 25 and 75%) and the median, whiskers represent the upper and lower value within 1.5 times the IQR. **(E)** Fold change of metabolic pathway activities in cell types in moderate vs. normal (left panel) and severe vs. normal (right panel). **(F)** Fold change of metabolic pathway activities in cell types in severe vs. moderate.

We downloaded the scRNA-seq data of BALF cells from the GEO database (GSE145926) of 3 moderate COVID-19 patients (P141, P142, and P144), 6 severe infected patients (namely, one severe patient [P143] and 5 critical patients [P145, P146, P148, P149, and P152]), and 3 HCs [P51, P52, and P100]. We reanalyzed the scRNA-seq data and marked the cells as 10 lineages identified by characteristic genes, consistent with the paper by Liao (22): T cells (*CD3D*), NK cells (*KLRD1*), B cells (*MS4A1* and *CD79A*), plasma cells (*XBP1* and *IGHG4*), mast cells (*CPA3*, *TPSAB1* and *TPSB2*), neutrophils (*FCGR3B*), macrophages (*CD68*), myeloid dendritic cells (mDCs) (*CD1C* and *CLEC9A*), plasmacytoid dendritic cells (pDCs) (*LILRA4*) and epithelial cells (*TPPP3* and *KRT18*). To study the roles of metabolic changes of immune cells in the BALF of COVID-19 patients, we screened 1,651 metabolism-related genes (involved in 63 signal pathways) from the GO and KEGG databases to analyze their regulation in immune cells. We first repeated the t-SNE analysis only based on the expression levels of 1,651 metabolic genes and found that the clustering patterns of these metabolic genes were similar to those of the genome-wide genes, which could clearly distinguish the above 10 cell types (**Figures 1A, B**, and **Supplementary Figure 1A**). This implied that the metabolic plasticity of cells could reflect the specific factors of different types of cells.

We scored each metabolic pathway to compare the characteristics of metabolic pathway variation between different cell types. Neutrophils showed lower metabolic activity, which may be related to the short life cycle of neutrophils and the low transcriptional activity (**Figure 1C**). In most cell types, COVID-19 patients have more active metabolic pathways (**Supplementary Figure 1B**); moderate patients had the highest overall metabolic pathway activity and there was no significant difference in the metabolic activity of severe patients compared with HCs (**Figure 1D** and **Supplementary Figure 1C**). COVID-19 patients showed significant remodeling of metabolic processes, namely, carbohydrate metabolism, lipid metabolism, amino acid metabolism, and nucleic acid metabolism. We calculated the metabolic pathway activities in the cell types with cell counts >20 in both HCs and patients. In both moderate and severe COVID-19 patients, nucleic acid metabolism, amino acid metabolism (phenylalanine, cysteine, methionine, arginine, proline and tyrosine), nicotinate and nicotinamide metabolism, folate biosynthesis, and glycolysis were generally upregulated in all cell types (**Figure 1E**). There was more significant upregulation in B cells of phenylalanine metabolism, glycine, serine, threonine and tyrosine metabolism, linoleic acid metabolism and cholesterol metabolism than in other cell types. In macrophages, carbohydrate metabolism such as the pentose phosphate pathway was upregulated in moderate patients, but downregulated in severe patients. The metabolic activity of severe patients was generally lower than that of moderate patients, but the levels of energy metabolism and amino acid metabolism such as glutamate, glutamine, glycine, serine, threonine, arginine and proline were higher than those of moderate patients (**Figure 1F**). Interestingly, most of the metabolic pathways of macrophages in severe patients showed a lower activity compared with other cell types (**Supplementary Figure 1D**). In summary, we constructed a global metabolic reprogramming map of BALF immune cells in COVID-19 patients.

### Metabolic Reprogramming of BALF T Cells in COVID-19 Patients

**Figure 2:**
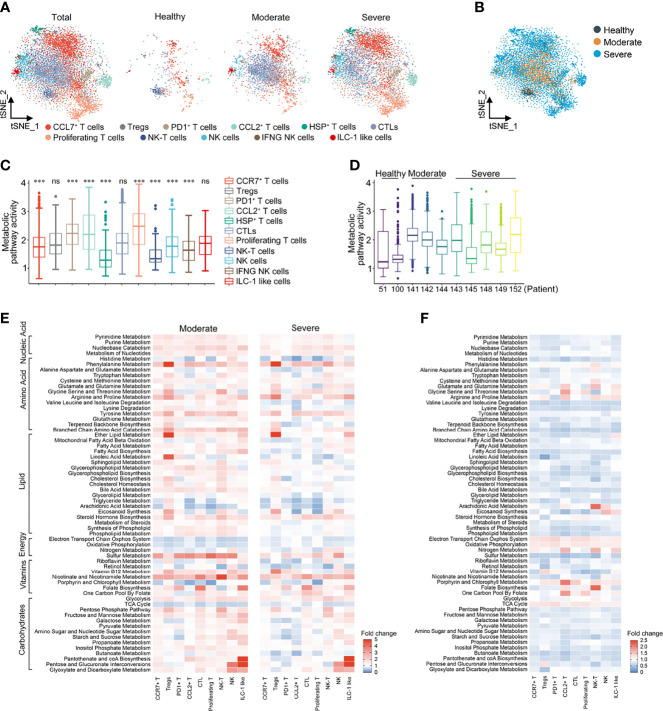
Metabolic reprogramming of T cells in COVID19 patients. **(A)** tSNE plots of metabolic gene expression profiles within each sample type, color-coded by T cell subsets. **(B)** tSNE plots of metabolic gene expression profiles, color-coded by sample type. **(C)** Distributions of metabolic pathway activities in different T cell subsets. The box plots were defined by the interquartile range (IQR, the range between the 25 and 75%) and the median, whiskers represent the upper and lower value within 1.5 times the IQR. The significance was performed by Wilcoxon rank sum test. ***P < 0.001, ns, not significant. **(D)** Distributions of metabolic pathway activities in different patients. The box plots were defined by the interquartile range (IQR, the range between the 25 and 75%) and the median, whiskers represent the upper and lower value within 1.5 times the IQR. **(E)** Fold change of metabolic pathway activities in T cell subsets in moderate vs. normal (left panel) and severe vs. normal (right panel). **(F)** Fold change of metabolic pathway activities in T cell subsets in severe vs. moderate.

T cells and NK cells were reanalyzed and labeled as 11 lymphocyte clusters based on the expression of canonical genes (**Supplementary Figures 2A, B**). There was a significant increase in the ratio of Tregs and NK cells in COVID-19 patients compared with HCs. In addition, compared with moderate patients, the proportions of Tregs, PD1^+^ T cells, CCL2^+^ T cells, NK cells and IFNG^+^ NK cells in severe patients were higher, while the proportions of CTL cells and ILC-like NK cells were lower (**Supplementary Figures 2C, D**), indicating that COVID-19 infection significantly disrupted the T cell immune response. The t-SNE reclustering analysis based on the expression levels of 1,651 metabolic genes could also clearly distinguish the above 11 T cell and NK cell clusters (**Figures 2A, B**).

Compared with T cells, the metabolic activity of NK cells was significantly downregulated, which was particularly obvious in HCs. Compared with HCs, the metabolic activity of PD1^+^ T cells, CTL cells and proliferating T cells in COVID-19 patients increased the most significantly (**Figure 2C** and **Supplementary Figure 3A**). The overall metabolic pathway activity of moderate and severe COVID-19 patients was both significantly higher than that of HCs, while the metabolic activity of moderate COVID-19 patients was significantly higher than that of severe patients (**Figure 2D** and **Supplementary Figure 3B**). Among the various cell clusters, nucleic acid metabolism, amino acid metabolism, lipid metabolism and carbohydrate metabolism in moderate patients were generally higher than that in HCs, but there was a cell specificity in severe patients. Compared with HCs, the levels of galactose, pyruvate and propanoate metabolism in PD1^+^ T cells and Tregs were significantly lower in moderate and severe patients, while glycerol lipid and arachidonic acid metabolism were significantly decreased in CCL2^+^ and proliferative T cells (**Figure 2E**). Compared with HCs, the activity of the electron transport chain system, oxidative phosphorylation and TCA cycle was reduced in patients with COVID-19, while glycolytic activity was increased, suggesting that the energy metabolism of patients with COVID-19 was dominated by anaerobic metabolism (**Figure 2E**). Consistent with the changes in the total cells, the energy metabolism level of severe patients was lower than that of moderate patients (**Figure 2F**). It is worth noting that these metabolic pathways in T cells and NK cells, but not in the total cells, could be distinguished between HCs, moderate patients and severe patients (**Supplementary Figure 3C**).

### Metabolic Reprogramming of BALF Macrophages in COVID-19 Patients

**Figure 3:**
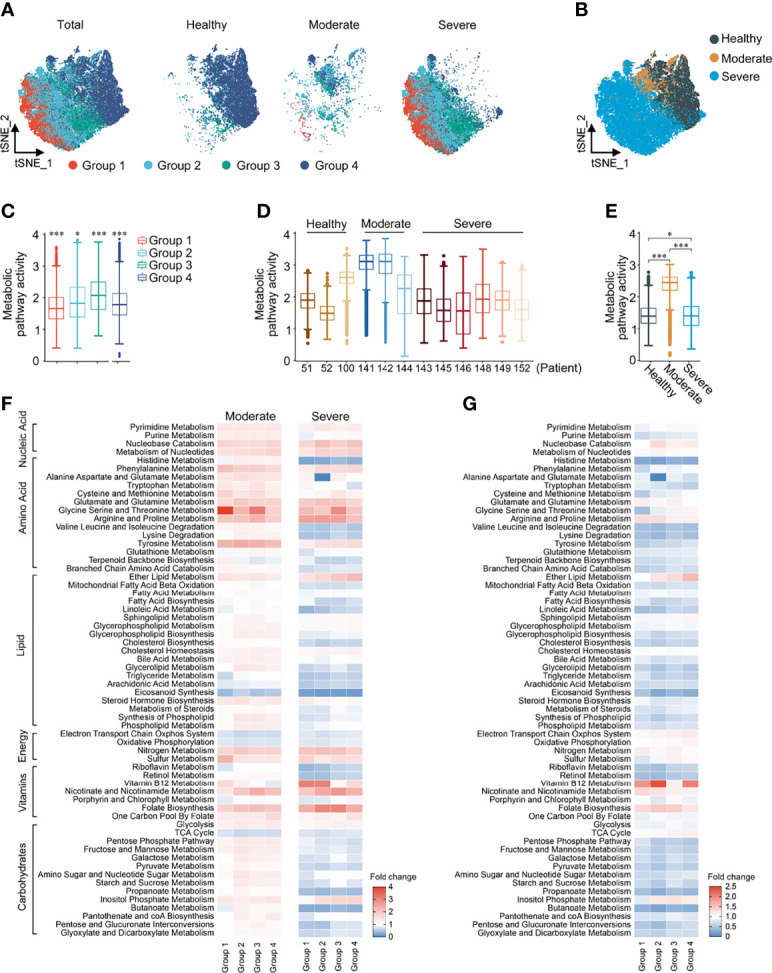
Metabolic reprogramming of Macrophages in COVID19 patients. **(A)** ttSNE plots of metabolic gene expression profiles within each sample type, color-coded by macrophage subsets. **(B)** tSNE plots of metabolic gene expression profiles, color-coded by sample type. **(C)** Distributions of metabolic pathway activities in different macrophage subsets. The box plots were defined by the interquartile range (IQR, the range between the 25 and 75%) and the median, whiskers represent the upper and lower value within 1.5 times the IQR. The significance was performed by Wilcoxon rank sum test. **(D)** Distributions of metabolic pathway activities in different patients. The box plots were defined by the interquartile range (IQR, the range between the 25 and 75%) and the median, whiskers represent the upper and lower value within 1.5 times the IQR. The significance was by Wilcoxon rank sum test. **(E)** Distributions of metabolic pathway activities in different sample types. The box plots were defined by the interquartile range (IQR, the range between the 25 and 75%) and the median, whiskers represent the upper and lower value within 1.5 times the IQR. The significance was performed by Wilcoxon rank sum test. **(F)** Fold change of metabolic pathway activities in macrophage subsets in moderate vs. normal (left panel) and severe vs. normal (right panel). **(G)** Fold change of metabolic pathway activities in macrophage subsets in severe vs. moderate. *P < 0.05, ***P < 0.001.

Similarly, the 4 macrophage groups [Group 1, FCN1^hi^; Group 2, FCN1^lo^SPP1^+^; Group 3, SPP1^+^; Group 4, FABP4^+^; consistent with the paper by Liao (22)] could be well distinguished by the expression of 1651 metabolic genes in t-SNE reclustering analysis (**Figures 3A, B**). The overall metabolic level of Group 3, marked as alternative M2-like macrophages (22), was the highest among the four groups (**Figure 3C**). Metabolic pathway activity of moderate COVID-19 patients was significantly higher than that of both HCs and severe COVID-19 patients (**Figures 3D, E**). In moderate patients, all signal pathways except aerobic metabolism and eicosanoid synthesis were upregulated compared with HCs. In severe patients, only nucleic acid metabolism and the metabolism of some amino acids were upregulated, and most lipid and carbohydrate metabolism levels were lower than those of HCs (**Figure 3F**). When we performed an unsupervised clustering analysis of macrophage groups based on the scores of metabolic pathways, we found that, surprisingly, macrophages in moderate patients had a closer metabolic profile that more closely resembled that of HCs than that of severe patients. The top half of the pathways were similar between HCs and moderate patients, while the bottom half was similar between severe and moderate patients. More importantly, unlike in total cells and T cells, the expression of metabolic pathways in macrophages could completely separate healthy person, moderate and severe patients (**Supplementary Figure 4**). Although the metabolic changes of macrophages in the four groups were basically the same in both moderate and severe patients, we could observe that in Group 2 of severe patients, the levels of alanine, aspartate and glutamate metabolism significantly decreased (**Figure 3G**). Based on these findings, we could conclude that in COVID-19 patients, metabolic reprogramming in T cell subclusters and macrophage subclusters could better reflect the differences between patients at different disease stages than in all immune cells.

### Cytokine Storm in COVID-19 Patients Was Correlated With Metabolic Reprogramming

**Figure 4:**
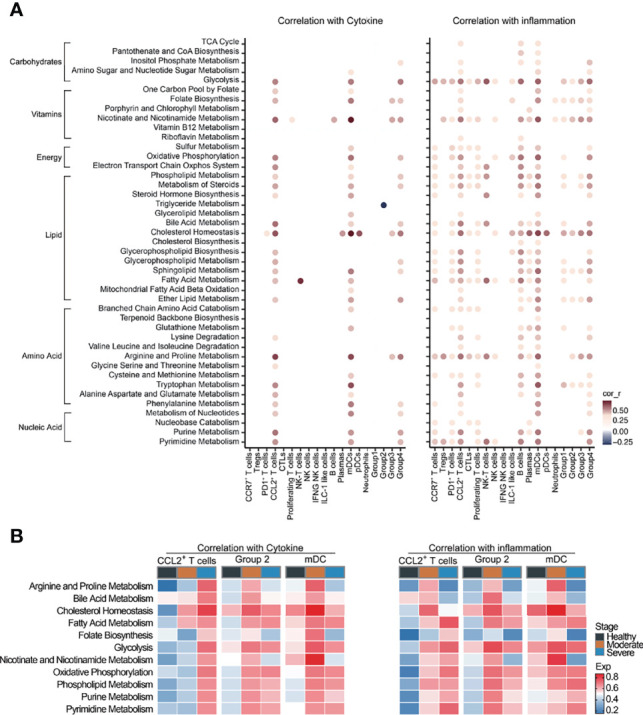
Cytokine storm was correlated with metabolic reprogramming in COVID-19 patients. **(A)** Dot-plot of correlation between metabolic pathway activities with cytokine score (left panel) and inflammation score (right panel). **(B)** Heatmap of correlation between metabolic pathway activities with cytokine score (left panel) and inflammation score (right panel).

Cytokine storms are often observed in COVID-19 patients, especially in severe patients. Based on the cytokine and inflammation related genes reported to be related to COVID-19, we performed cytokine score and inflammation score analysis on each cell cluster to evaluate the potential contribution of each cell cluster to the cytokine storm. The cytokine and inflammation scores in patients were significantly increased (**Supplementary Figure 5A**). CCL2^+^ T cells, mDCs and macrophages (Groups 1–4) had significantly higher cytokine and inflammation scores compared with other clusters (**Supplementary Figure 5B**). Cytokine and inflammation scores in these six cell clusters of COVID-19 patients were significantly increased, especially in severe stages (**Supplementary Figure 5C**), suggesting that these cells might play a central role in driving the cytokine storm. We next calculated the correlations between metabolic pathways and the cytokine and inflammation scores in each cell clusters to investigate the impact of metabolic reprogramming on the cytokine storm of COVID-19 patients. We screened the interactions whose correlation coefficient was greater than 0.3 or less than −0.3, and the corrected *P*-value was less than 0.05. Glycolysis, folate biosynthesis, nicotinate and nicotinamide metabolism, oxidative phosphorylation, lipid metabolism (such as phospholipid, bile acid, cholesterol and fatty acid), arginine and proline metabolism and pyrimidine and purine metabolism were positively correlated with the cytokine and inflammation scores in CCL2^+^ T cells, mDCs and Group 2 macrophages (**Figure 4A**). Strong correlations between the cytokine and inflammation scores and metabolic pathways were observed in COVID-19 patients, especially in severe patients, and weak correlations were observed in HCs (**Figure 4B**). Especially in CCL2^+^ cells, the correlation in severe patients between these pathways and the cytokine score was significantly higher than in moderate patients and HCs, indicating that these pathways might be activated after SARS-CoV-2 infection, affecting the metabolic reprogramming of immune cells and generating a cytokine storm (**Figure 4B**). These findings demonstrate that metabolic changes in immune cells might play important roles in the formation of cytokine storm in COVID-19 patients.

### Compass Analysis of BALF scRNA-seq Data in COVID-19 Patients

**Figure 5:**
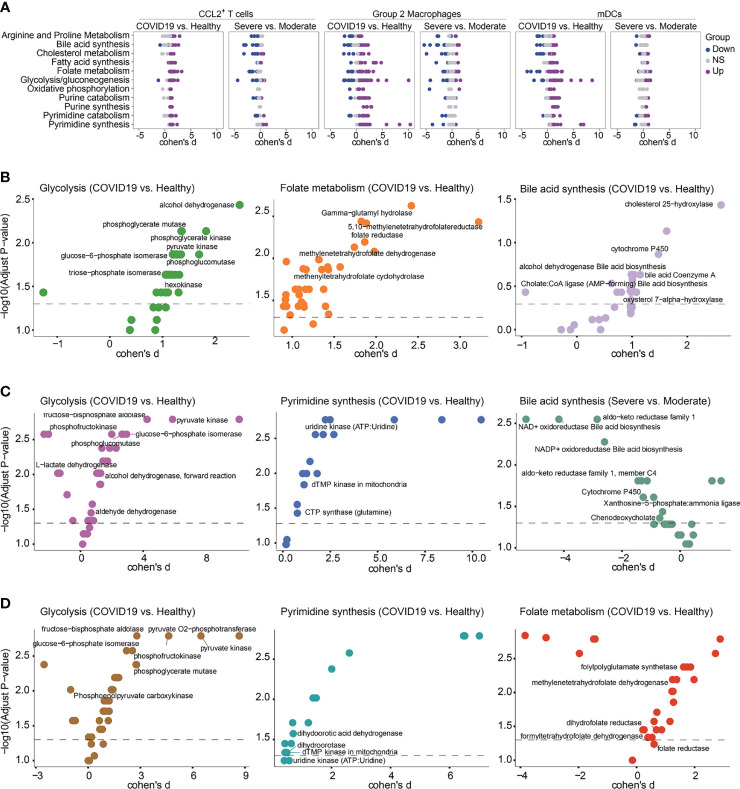
Metabolic reprogramming of CCL2^+^ T cells, mDCs and Group2 Macrophages analyzed by Compass. **(A)** Differential activity of metabolic reactions. **(B)** Compass-score differential activity test in CCL2^+^ T cells. **(C)** Compass-score differential activity test in Group 2 Macrophages. **(D)** Compass-score differential activity test in mDCs.

In order to verify the results of our metabolic analysis, we used another metabolic calculation method, Compass, to analyze the metabolic status of CCL2^+^ T cells, Group 2 macrophages and mDCs, which were the important contributors to the cytokine storms. Compass analysis was conducted based on the Recon2 database, which covered 7,440 reactions and 2,626 metabolites (23, 24). In Compass analysis, the overall metabolic state of the cell is quantitatively analyzed by calculating the score of each reaction in each cell. Consistent with previous results, the metabolic pathways in **Figure 5A** were upregulated in COVID-19 patients compared with HCs both in CCL2^+^ T cells, Group 2 macrophages and mDCs. Although bile acid synthesis was increased in COVID-19 patients, in CCL2^+^ T cells and Group 2 macrophages, compared with moderate patients, the metabolic responses related to bile acid synthesis were almost all downregulated in severe patients (**Figure 5A**). Compass can help us examine changes in metabolism at the level of a single reaction rather than the entire pathway (**Figures 5B**–**D**). Although the same metabolic pathway was elevated in a variety of cells, the expression status of each of these responses remained cell-specific. For example, the glycolytic pathway was upregulated in CCL2^+^ T cells (severe vs. moderate patients) and in macrophages and mDCs (COVID-19 patients vs. HCs). Compass predicted that key enzymes such as pyruvate kinase and glucose-6-phosphate isomerase were all upregulated in three cell types, while the upregulation of phosphoglycerate mutase was more pronounced in CCL2^+^ T cells and mDCs. The downregulation of L-lactate dehydrogenase was only observed in Group 2 macrophages. The upregulation of folate metabolism was seen in both CCL2^+^ T cells and mDCs, but the upregulation of folate reductase was statistically significant only in CCL2^+^ T cells and not in mDCs.

Furthermore, to validate our description of the metabolic profile of COVID-19 BALF samples, we performed metabolic analysis of immune cells in independent BALF scRNA-seq data from 3 moderate and 9 severe COVID-19 patients, and we selected CCL2^+^ T cells, Group 2 macrophages and mDCs for key analysis using Compass (**Supplementary Figures 6A, B**). Highly consistent with the above analysis results, overall, the metabolic pathways were generally downregulated in severe patients compared with moderate patients. Arginine and proline, glycine serine and threonine, glutamate and glutamine metabolism, oxidative phosphorylation and electron transport chain system were elevated in all cell types, while folate biosynthesis and vitamin B12 metabolism were increased in neutrophils, mono-macrophages and mDCs (**Supplementary Figure 6A**). Compass analysis of CCL2^+^ T cells, Group 2 macrophages and mDCs was also consistent with **Figure 5A**.

### Metabolic Reprogramming of Nasopharyngeal Swab Immune Cells in COVID-19 Patients

**Figure 6:**
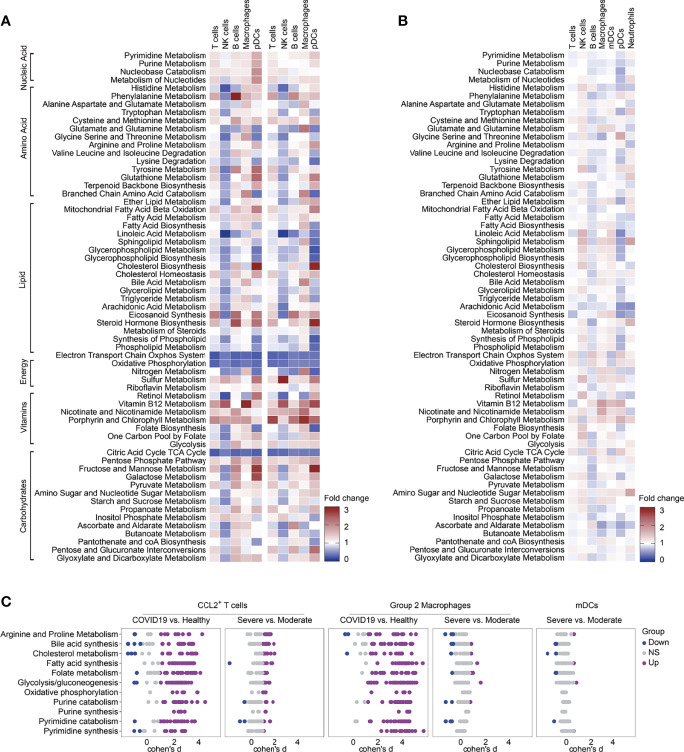
Metabolic reprogramming of immune cells in independent pooled nasopharyngeal/pharyngeal swabs scRNA sequencing data. **(A)** Fold change of metabolic pathway activities in cell types in moderate vs. normal (left panel) and severe vs. normal (right panel). **(B)** Fold change of metabolic pathway activities in cell types in severe vs. moderate. **(C)** Differential activity of metabolic reactions.

Finally, we verified the expression levels of metabolic pathways in independent nasopharyngeal swab scRNA-seq data from 19 COVID-19 patients and 5 HCs. Consistent with previous results from BALF, nicotinate and nicotinamide metabolism, glycolysis and pyrimidine synthesis were elevated in COVID-19 patients. Similar to the results from BALF, the electron transport chain system, oxidative phosphorylation, and the citric acid cycle were significantly downregulated in all analyzed cell types in COVID-19 patients (**Figures 1E**, **6A**). We found that most of the pathways are downregulated in NK cells in nasopharyngeal swabs from COVID-19 patients compared with those from HCs (**Figure 6A**). It is interesting to notice that almost all of the metabolic pathways are downregulated in BALF from severe COVID-19 patients compared with that from moderate ones (**Figure 1F**), whereas the phenomenon is obscure when comparing the nasopharyngeal swabs from severe and moderate COVID-19 patients (**Figures 1F**, **6B**). However, vitamin B12 metabolism and nicotinate and nicotinamide metabolism are all significantly upregulated in macrophages in both analyses (**Figures 1F**, **6B**). Then we analyzed the metabolic pathways within CCL2^+^ T cells, group 2 Macrophages, and mDCs between different groups in nasopharyngeal swabs using Compass analysis. We found that most of the metabolic reactions within 11 metabolic pathways are upregulated in CCL2^+^ T cells and Group 2 macrophages in COVID-19 patients compared with those from HCs, but metabolic reaction activity is similar in CCL2^+^ T cells, Group 2 macrophages, and mDCs from severe and moderate COVID-19 patients (**Figure 6C**). Then we analyzed the metabolic pathways within CCL2^+^ T cells, Group 2 macrophages, and mDCs between different groups in nasopharyngeal swabs using Compass analysis. We found that most of the metabolic reactions within 11 metabolic pathways are upregulated in CCL2^+^ T cells and Group 2 macrophages in COVID-19 patients compared with HCs, but metabolic activity is similar in in CCL2^+^ T cells, Group 2 macrophages, and mDCs in severe and moderate COVID-19 patients (**Figure 6C**). Given the presence of mDC populations in only one HC sample in these nasopharyngeal swab data, we had no means to perform a statistical analysis of the differences in mDC metabolism between HCs and COVID-19 patients. In conclusion, in the nasopharyngeal swab samples, unlike the BALF samples, there were no significant differences in metabolic activity between severe and moderate patients. In our study, we found that almost all metabolic pathways are downregulated in BALF from severe COVID-19 patients compared with that from moderate COVID-19 patients, but the same phenomenon was not observed in nasopharyngeal swabs. The virus infects person from the upper respiratory system to the lower respiratory system, and finally causes the pneumonia. During the development of pneumonia from the moderated to the severe state, most of the destructive events might take place in the lower respiratory system, explaining why the downregulation of metabolic pathways was more prominent in BALF than in nasopharyngeal swabs.

## Discussion

As the characteristics of peripheral immune cells are different from those in lungs, with respect to both quality characteristics and duration of the immune response, the analysis of the peripheral immune metabolic reprogramming in COVID-19 might not be comprehensive. Therefore, there was a need to better understand the metabolic landscape in BALF of COVID-19 patients. Previous multi-omics metabolism studies have found a dysregulation of the TCA cycle, fructose and mannose metabolism, tryptophan metabolism, glycolysis and gluconeogenesis in COVID-19 patients. However, there is a lack of understanding of cell-specific metabolic reprogramming, especially in *in situ* immune cells in lung tissue. This study comprehensively explored and scored the transcriptional changes of genes in 63 metabolic pathways in 22 cell types in BALF and provided evidence of immune metabolic reprogramming in BALF immune cells in patients with COVID-19. In severe COVID-19 patients, the exhaustion of immune cells resulted in lymphocytopenia and impaired immune functions, and also the overall metabolic activity of immune cells showed a decreasing trend.

We have observed that the upregulation of glycolytic pathways in COVID-19 patients occurs in all immune cell types. Although these immune cells did not express ACE2 or TMPRSS2, SARS-CoV-2 RNAs was detected in a diverse set of immune cells, namely, neutrophils, macrophages, plasma B cells, T cells, and NK cells (10, 25). Studies indicated that viruses entering the host cells induced metabolic reprogramming of host cells by triggering mitochondrial reactive oxygen species (ROS) production, which induced stabilization of hypoxia-inducible factor-1α (HIF-1α) and consequently promoted glycolysis. The increase of glycolysis also can be activated independent of the infection itself, such as a direct response to hypoxia (26). Enhanced glycolysis could sustain the proliferation and activation of T cells, B cells and M1 macrophages (27). At the same time, we found that these enhanced glycolysis reactions were also related to the generation of cytokine storms.

Consistent with B cells in PBMCs, metabolism of alanine, aspartate, glutamate and lysine was downregulated in B cells in BALF of patients with COVID-19. However, fatty acid biosynthesis, which displayed enhanced activity in PBMC B cells (28), was increased in moderate patients and decreased in B cells in BALF from severe patients. Phospholipid synthesis and protein modification, which are necessary for transcription and translation during B cell proliferation, differentiation, and immunoglobulin synthesis, require fatty acids as precursors (29). The increase in fatty acids and cholesterol supported the doubling of membrane content associated with proliferation and the formation of the endomembrane immunoglobulin secretion network (30). Previous studies showed that B cells in PBMCs of COVID-19 patients highly expressed the gene encoding immunoglobulin, suggesting that they played a role in the secretion of antigen-specific antibodies. Especially the sera of severe patients have high titers of SARS-CoV-2 specific antibodies (10, 31). This explains why the fatty acid synthesis in B cells of COVID-19 patients was significantly increased. In severe patients, a significant loss of the chemokine receptor CXCR5 in B cells caused damage to the germinal center response and the dysregulates humoral immune response, and a large number of transcripts related to B cell function were downregulated (32–34). This could explain why the synthesis of fatty acids in the B cells in severe patients was reduced in BALF of severe patients.

Studies have reported that compared with HCs, the glycolysis, the TCA cycle and oxidative phosphorylation activity of peripheral blood T cells in COVID-19 patients were all increased (28), while we have observed that in BALF, the electron transport chain system, oxidative phosphorylation and the TCA cycle were decreased, and only glycolytic activity was increased. The electron transport chain system, oxidative phosphorylation and the TCA cycle are all reactions completed in the cell mitochondria. In severe patients, cytokines, such as TNF-α and IL-6, with a sharply elevated content in BALF could hinder mitochondrial oxidative phosphorylation and ATP production, leading to mitochondrial membrane permeabilization and changes in mitochondrial dynamics, resulting in serious mitochondrial damage and further lung damage (35, 36). Although previous studies reported that immune cells in peripheral blood also have mitochondrial damage (37, 38), we might infer that in the BALF of COVID-19 patients, especially in severe patients, the mitochondrial damage of immune cells in the lung bronchus region was more severe than that in the peripheral circulation.

Ren et al. proposed that monocyte subsets might contribute to cytokine storms in PBMCs (10). Similarly, we found that in infected tissue, CCL2^+^ T cells, Group 2 macrophages characterized by high expression of SPP1 and mDCs might also be the main sources of cytokine storm. mDCs and CCL2^+^ T cells were distributed in each disease stage, and the proportion of Group 2 macrophages increased in severe patients, and the cytokine and inflammation scores were all significantly elevated in cell types in severe patients. These three cell types contribute to the cytokine storm *via* enhanced cell ratios, enhanced expression of inflammatory molecules, or both in severe COVID-19 patients. The link between metabolism and inflammatory responses is considered to be an important pathway for regulating cytokine storms. Both activation and differentiation of T cells are highly correlated with metabolic reprogramming. Metabolic reprogramming of macrophage results in a high dependence of cellular energy metabolism on glycolysis to meet the high demands of macrophages to fight infection in an inflammatory environment. DC activation is often accompanied by upregulation of glucose uptake and increased fatty acid synthesis (39–41). In Group 2 macrophages of COVID-19 patients, two metabolic analysis methods both showed that glycolysis, fatty acid metabolism, bile acid synthesis and purine and pyrimidine metabolism were upregulated and strongly correlated to the cytokine and inflammation scores. This might provide us with insight into the factors regulating the production of cytokine storm at the metabolic level. Researchers have found that melatonin could inhibit the production of cytokine storm induced by COVID-19 by inhibiting the glycolysis in immune cells (42). How the other metabolic pathways affect the generation of cytokine storms and corresponding treatment strategies remain to be investigated.

In conclusion, we have systematically drawn and assessed the metabolic landscape of *in situ* immune cells in infected lung tissue obtained from BALF during SARS-CoV-2 infection. Enhanced glycolysis was the most important common metabolic feature of all immune cells in COVID-19 patients. Group 2 macrophages with high SPP1 expression were among the main contributors to the cytokine storm produced by infected lung tissue. Glycolysis, fatty acid metabolism, bile acid synthesis and purine and pyrimidine metabolism might be key metabolic factors regulating the production of the large quantities of cytokines in this group. This metabolic reprogramming in immune cells plays an important role in the progression of COVID-19 disease and the generation of cytokine storms.

## Data Availability Statement

The datasets presented in this study can be found in online repositories. The names of the repository/repositories and accession number(s) can be found in the article/**Supplementary Material**.

## Author Contributions

MMS and MS collected the data and performed data analysis. JD, XBP and BBG revised the figures and tables. MMS and FSY conceived the idea, supervised the study, critically revised the paper and guarantee the integrity of the study. All authors listed have made a substantial, direct, and intellectual contribution to the work and approved it for publication.

## Funding

This work was supported by a grant from the National Natural Science Foundation of China (31700790).

## Conflict of Interest

The authors declare that the research was conducted in the absence of any commercial or financial relationships that could be construed as a potential conflict of interest.

## Publisher’s Note

All claims expressed in this article are solely those of the authors and do not necessarily represent those of their affiliated organizations, or those of the publisher, the editors and the reviewers. Any product that may be evaluated in this article, or claim that may be made by its manufacturer, is not guaranteed or endorsed by the publisher.
