# Supplementary material for: Metabolic Landscape of Bronchoalveolar Lavage Fluid in Coronavirus Disease 2019 at Single Cell Resolution
Source: Front Immunol. 2022 Mar 8;13:829760. doi: 10.3389/fimmu.2022.829760 (PMC8957829; doi:10.3389/fimmu.2022.829760)
Supplement: Supplementary file 1 [file DataSheet_1.docx]

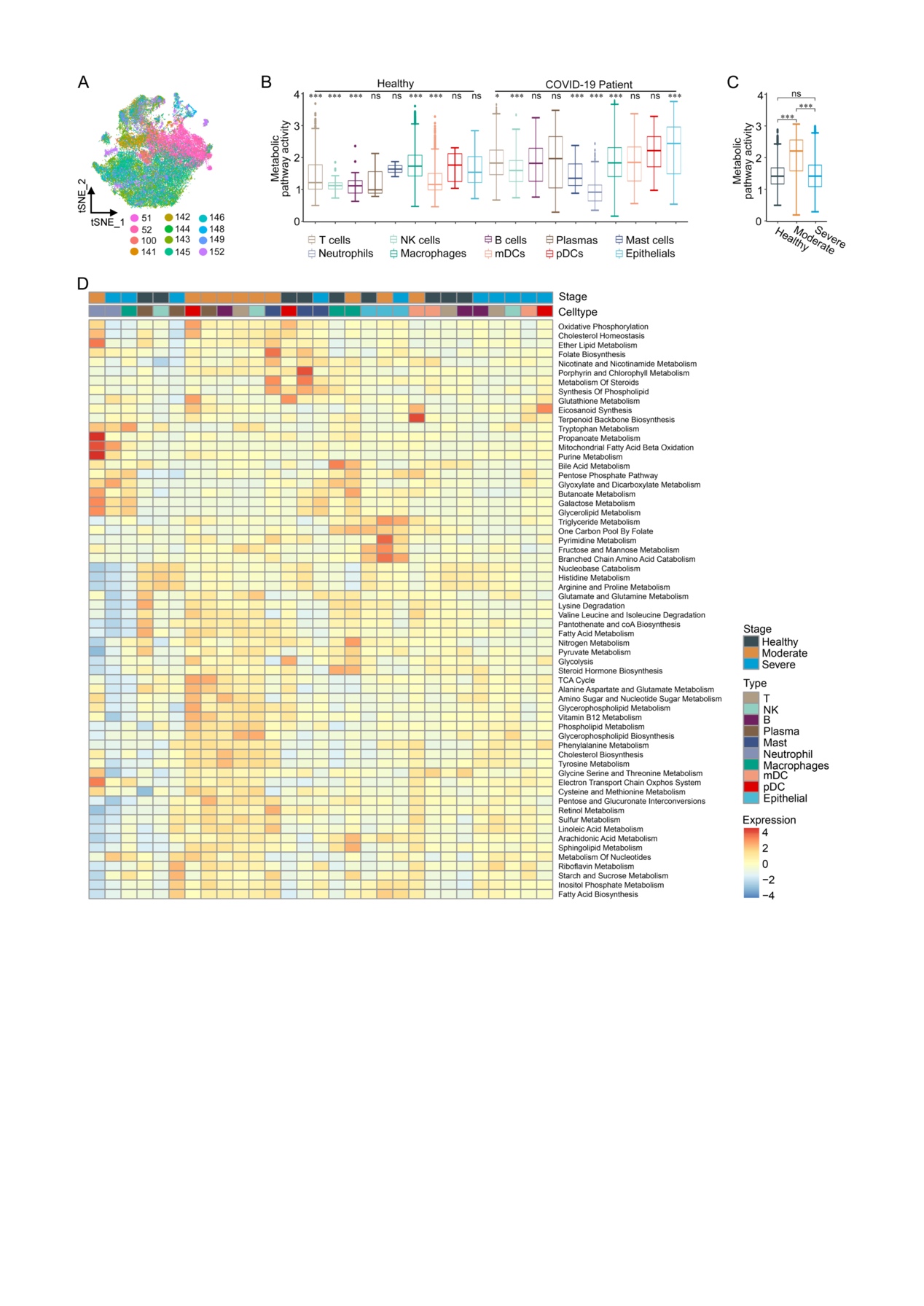


**Supplementary Figure 1. Metabolic reprogramming of immune cells in COVID19 patients.**

**A**, tSNE plots of metabolic gene expression profiles, color-coded by Seurat clusters. **B**, Distributions of metabolic pathway activities in different cell types. The box plots were defined by the interquartile range (IQR, the range between the 25% and 75%) and the median, whiskers represent the upper and lower value within 1.5 times the IQR. The significance was by Wilcoxon rank sum test. **C**, Distributions of metabolic pathway activities in different cell types. The box plots were defined by the interquartile range (IQR, the range between the 25% and 75%) and the median, whiskers represent the upper and lower value within 1.5 times the IQR. The significance was by Wilcoxon rank sum test. **D**, Heatmap of metabolic pathway activities in cell types. **P* < 0.05, ****P* < 0.001, ns, not significant.


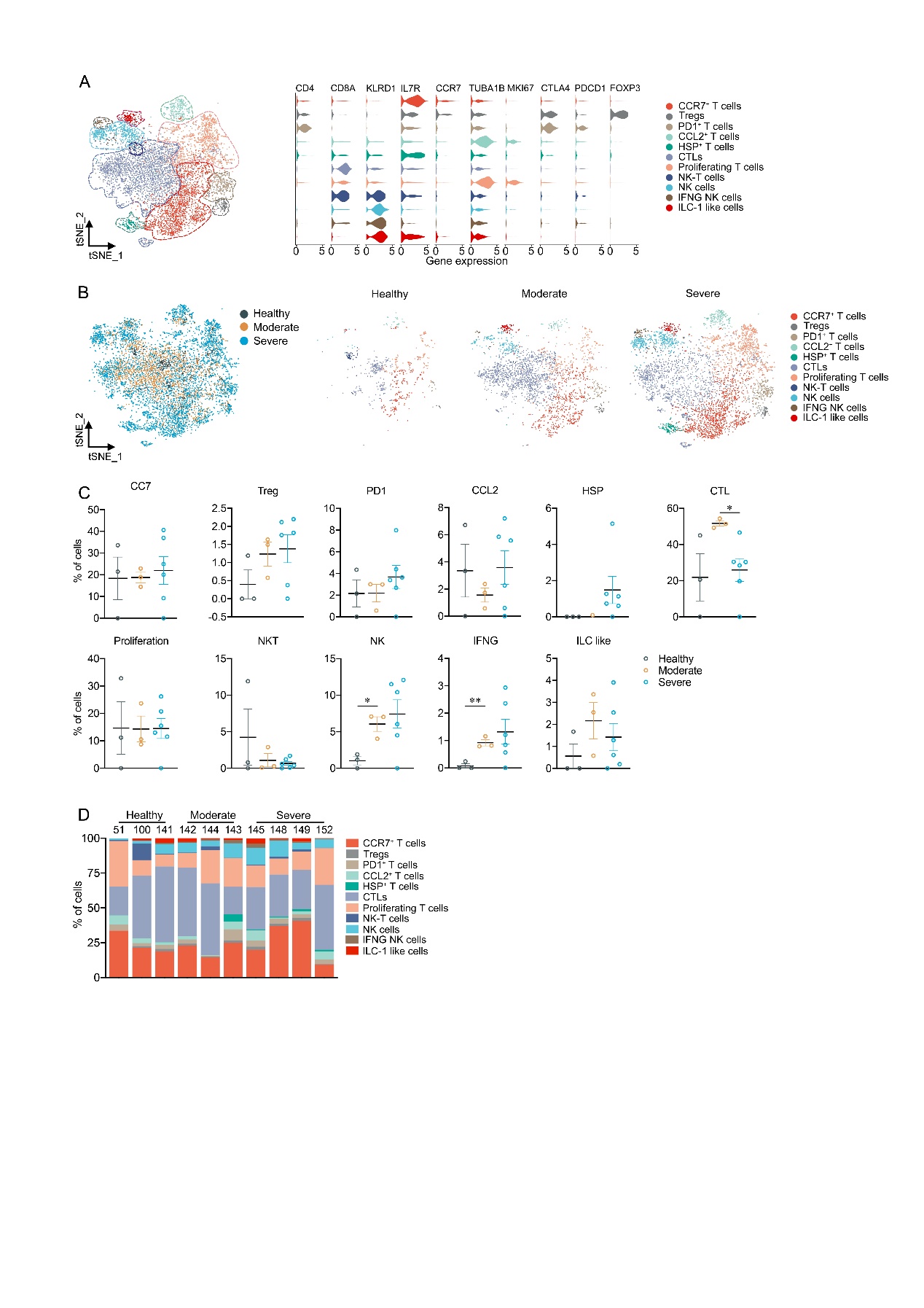


**Supplementary Figure 2. Dissection and clustering of T cells.**

**A**, tSNE plots (left panel) and marker genes (right panel) of T cells, color-coded by T cell subsets. **B**, tSNE plots of T cells within each sample type, color-coded by T cell subsets. **C**, Frequencies of T cell subsets according to the t-SNE plot. The significance was by Wilcoxon rank sum test. **P* < 0.05, ***P* < 0.01. **D**, Average proportion of each cell subset derived from each patient.


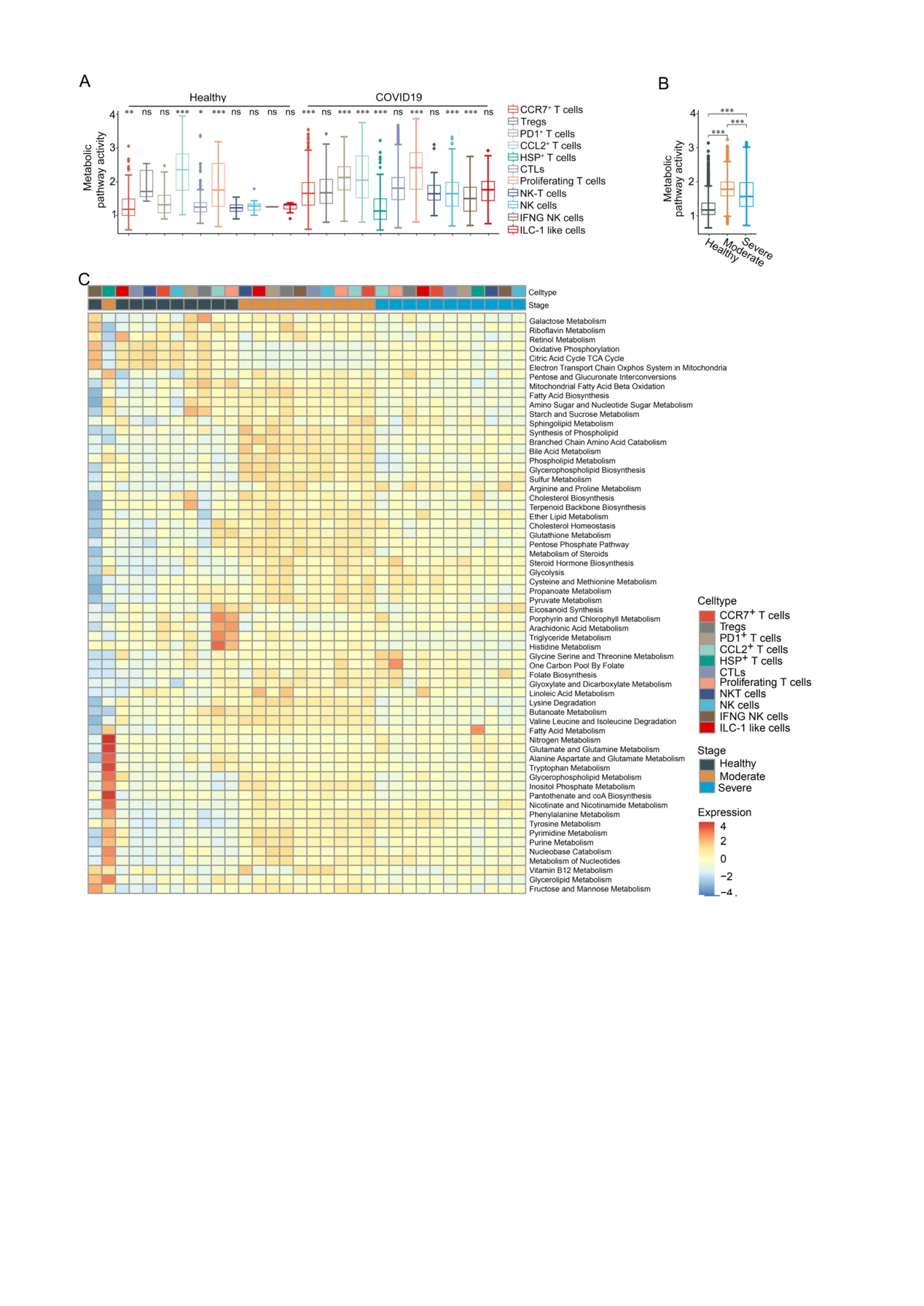


**Supplementary Figure 3. Metabolic reprogramming of T cells in COVID19 patients.**

**A**, Distributions of metabolic pathway activities in different cell types. The box plots were defined by the interquartile range (IQR, the range between the 25% and 75%) and the median, whiskers represent the upper and lower value within 1.5 times the IQR. The significance was by Wilcoxon rank sum test. **B**, Distributions of metabolic pathway activities in different cell types. The box plots were defined by the interquartile range (IQR, the range between the 25% and 75%) and the median, whiskers represent the upper and lower value within 1.5 times the IQR. The significance was by Wilcoxon rank sum test. **P* < 0.05, ***P* < 0.01, ****P* < 0.001, ns, not significant. **C**, Heatmap of metabolic pathway activities in cell types.


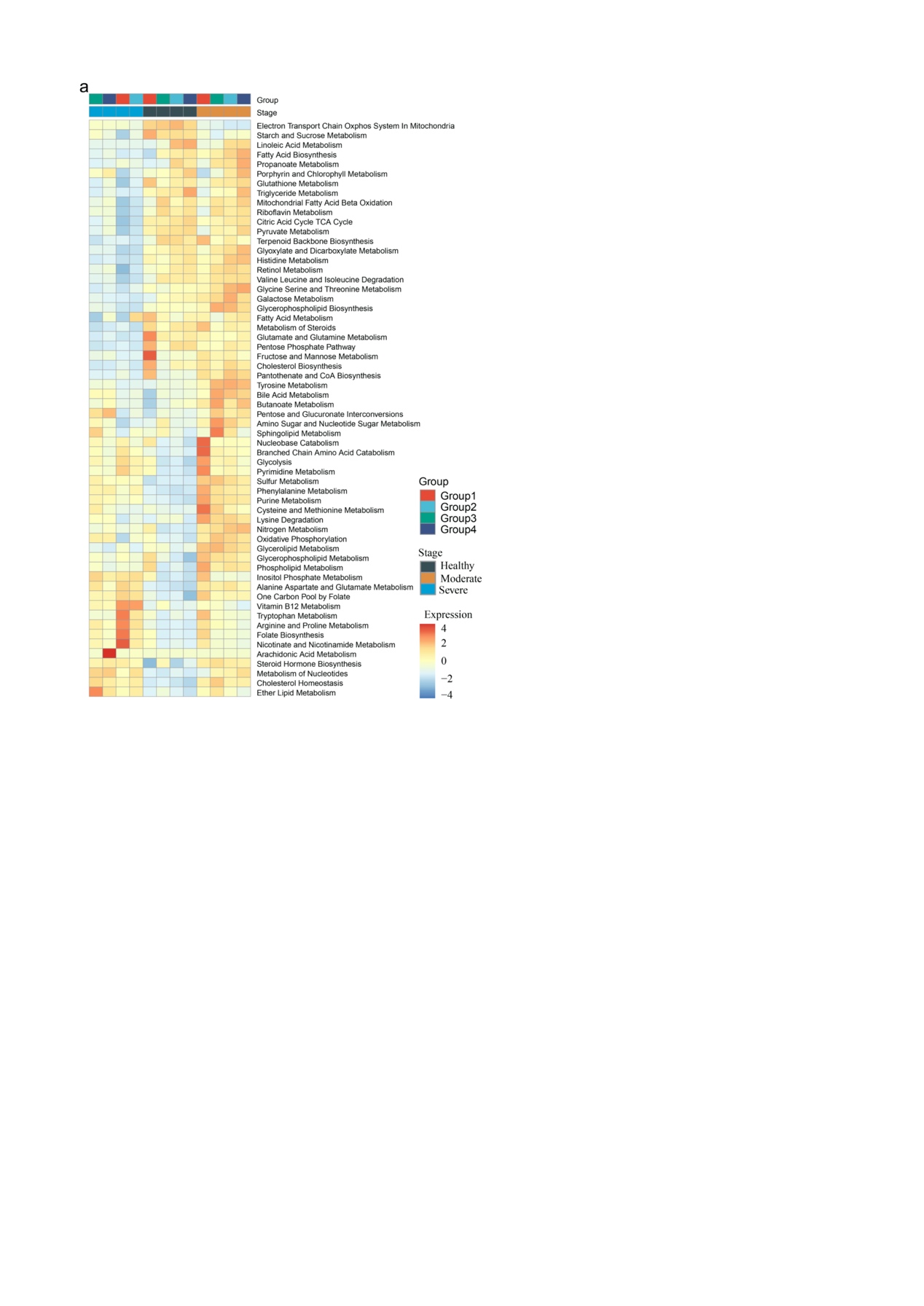


**Supplementary Figure 4. Metabolic reprogramming of Macrophages in COVID19 patients.** Heatmap of metabolic pathway activities in cell types.


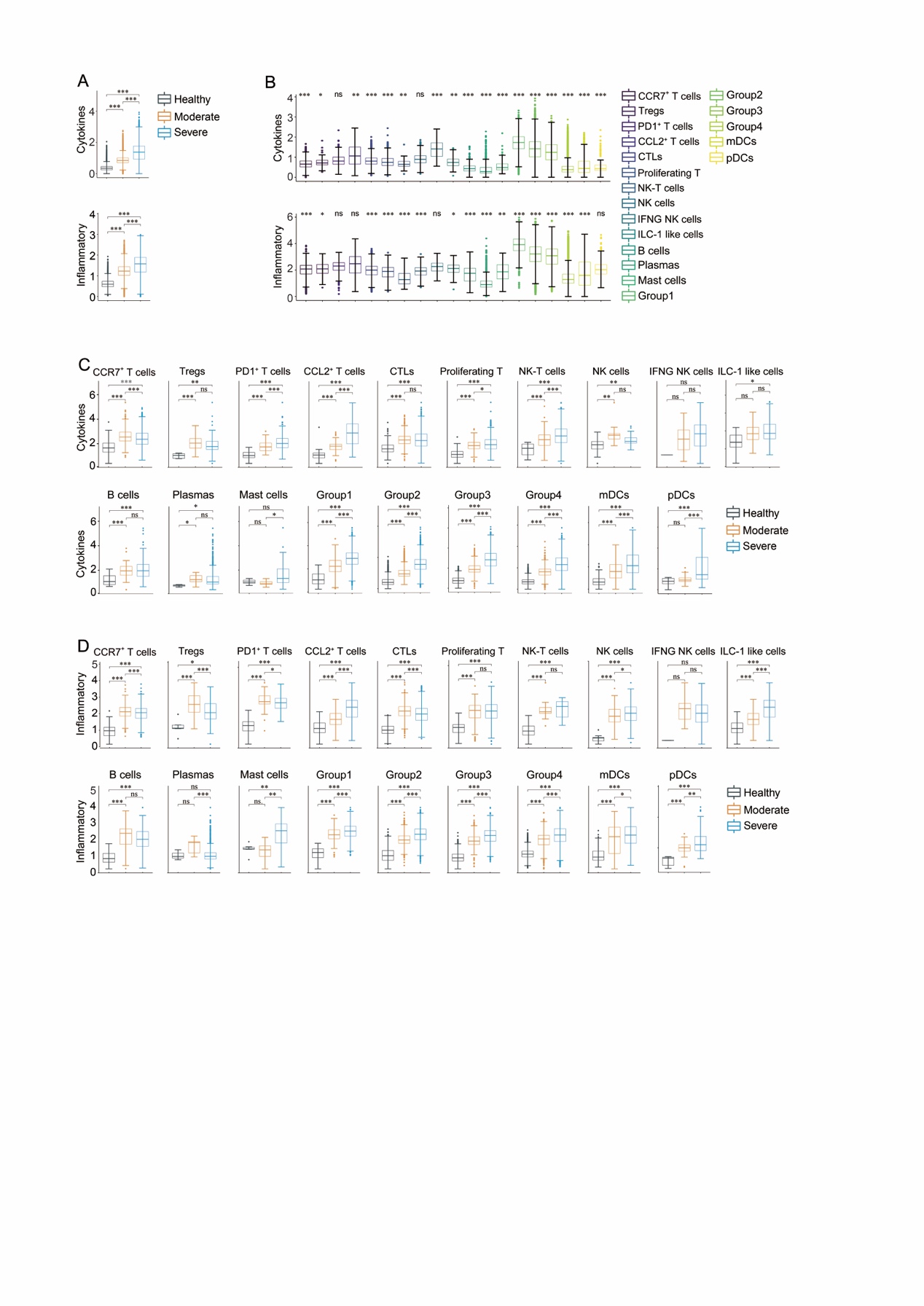


**Supplementary Figure 5. Cytokine storm in COVID19 patients.**

**A**, Distributions of cytokine score and inflammation score in different samples types. **B**, Distributions of cytokine score and inflammation score in different cell types. **C**, Distributions of cytokine score in different samples types in different cell types. The significance was by Wilcoxon rank sum test. **D**, Distributions of inflammation score in different samples types in different cell types. The significance was by Wilcoxon rank sum test. The box plots were defined by the interquartile range (IQR, the range between the 25% and 75%) and the median, whiskers represent the upper and lower value within 1.5 times the IQR. **P* < 0.05, ***P* < 0.01, ****P* < 0.001, ns, not significant.


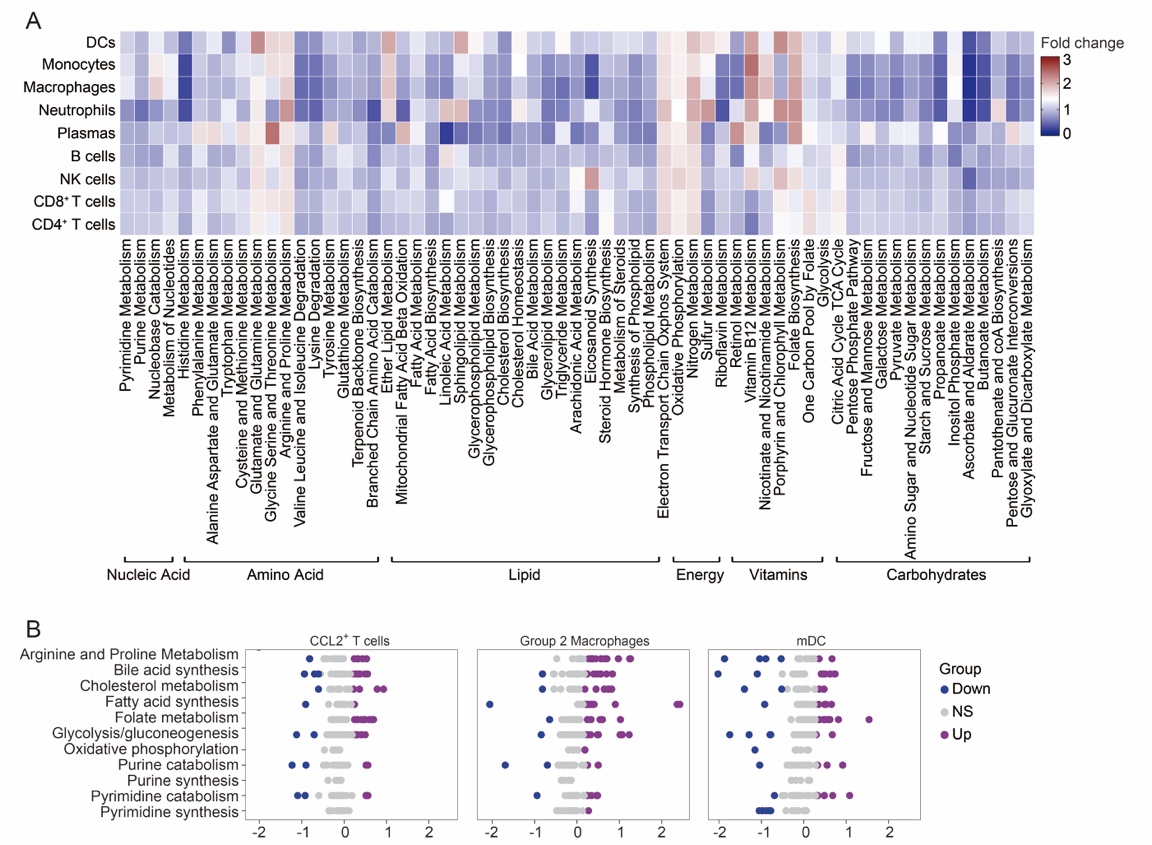


**Supplementary Figure 6. Metabolic reprogramming of immune cells in independent bronchoalveolar lavage fluid scRNA sequencing data.**

A, Fold change of metabolic pathway activities in cell types in severe vs. moderate. B, Differential activity of metabolic reactions.
